# Discovery of the First Neurotransmitter Receptor: The Acetylcholine Nicotinic Receptor

**DOI:** 10.3390/biom10040547

**Published:** 2020-04-03

**Authors:** Jean-Pierre Changeux

**Affiliations:** Department of Neuroscience, CNRS UMR 3571, Institut Pasteur & Collège de France, 75015 Paris, France; changeux@noos.fr

**Keywords:** pharmacological receptor, nicotinic acetylcholine receptor, first identification of a receptor, history of neuroscience

## Abstract

The concept of pharmacological receptor was proposed at the turn of the 20th century but it took almost 70 years before the first receptor for a neurotransmitter was isolated and identified as a protein. This review retraces the history of the difficulties and successes in the identification of the nicotinic acetylcholine receptor, the first neurotransmitter receptor to be identified.

The human brain is the most complex organ of the body which cannot be simply regarded as an electrophysiological machine or an electronic computer. Understanding the chemistry of the brain is a prerequisite for the understanding of its functions, in particular the cognitive ones and their pathological alterations. All the operations carried by the billions of its nerve cells and synapses are bound together by chemical signals which mediate information processing in the brain from the molecular to the cognitive level. Neurons produce and release molecules referred to as neurotransmitters, growth factors and/or hormones. These molecules are sent from one cell to another in small packages that deliver their cargo to the right place at the right time in the brain. Fundamental discoveries have been made in the past decades on the precise mechanism of the action of such cargo on their target cells, in other words the *signal transduction* they bring about. Signal transduction is the basic molecular process which mediates the conversion of a signal from outside the cell to a functional change in and/or within the cell. In all instances, the chemical signal binds to a particular molecule, a receptor, present in the cell membrane. This interaction causes a change in the functional properties of the receptor: the opening of an ion channel with the relevant electrical response (ligand-gated ion channels) *or* the activation of an intracellular cascade of enzymatic reactions eliciting a set of intracellular reactions (see G-protein coupled receptors (GPCRs), tyrosine kinase receptors).

Here, I would like to briefly recapitulate the discovery and biochemical identification of the first receptor for a neurotransmitter in the nervous system ever isolated as a protein. This receptor happened to be linked to an ion channel: the acetylcholine nicotinic receptor (nAChR). This discovery has introduced a major paradigmatic change in our understanding of the brain as a signal processing system opening novel avenues for the comprehension of neurological and psychiatric diseases and their pharmacology.

## 1. The Concept of Pharmacological Receptor

In his 1857 lecture “Lessons on the Effects of Toxic Substances and Drugs” [1] at the Collège de France Claude Bernard dealt with the physiological effects of the plant alkaloid curare and showed that curare does not alter muscle contraction but affects the peripheral action of the motor nerves on the muscle. This was the first localization of the action of curare but Bernard did not further specify adequately its precise target. Paul Ehrlich [2], concerned by the interaction of toxins with “antitoxic antibodies,” wrote that the “capacity to bind the antibodies must be related to the existence of specific atomic groupings which belong to the toxic complex, display a maximal specific affinity for a determined atomic grouping of the antitoxic complex and easily inserts in it, as a key and a lock according to the known analogy by Emil Fischer.” The theoretical notion of neurotransmitter receptor as we use it today belongs to John Newport Langley who in 1905-8 showed in the fowl that nicotine causes first a contraction (it acts as an agonist), then a block (the response desensitizes). On the other hand, curare blocks the effect of nicotine (as a competitive antagonist). Since none of these compounds prevented the contraction of the muscle, Langley concluded that “the muscle substance which combines with nicotine and curare is not identical with the substance which contracts. It is convenient to have a term for the especially excitable constituent, and I have called it *the receptive substance”* [3].

## 2. The Pioneers in the Unsuccessful Attempts to Identify the Nicotinic Acetylcholine Receptor

Carlos Chagas Filho (10 September 1910–16 February 2000) was born in Brazil as the second son of the Brazilian sanitary physician Dr. Carlos Chagas (1879–1934) who discovered American trypanosomiasis, also called Chagas disease. He studied medicine (1926) and became a professor at the Federal University of Rio de Janeiro. He contributed to the elucidation of the anatomy, physiology and pharmacology (effect of curare) of the electroplaque of the electric eel (*Electrophorus electricus*) and made early—unsuccesful—attempts to isolate the nAChR [4].

Eduardo De Robertis (11 December 1913–31 May 1988) was born in Buenos Aires and graduated in medicine from the University of Buenos Aires in 1939. He then went to Massachusetts Institute of Technology, where he described the neuro-tubules (1946–1949) and then to Seattle where he elucidated along with Henry S. Bennett the ultra-structure of synapses in sympathetic ganglia of frogs and in April 1954, made the seminal discovery of synaptic vesicles in nerve terminals (separately announced by Palade and Palay) [5]. He also made some apparently unsuccessful attempts to isolate several neurotransmitter receptors as proteolipids in non-aqueous solutions (see below). 

David Nachmansohn (17 March 1899–2 November 1983) was born in Dniepropetrovsk, (Ukraine), and moved to the Kaiser-Wilhelm Intitute fur Biologie in Berlin in 1926 where he worked with Otto Meyerhof and discovered that rapidly contracting muscles contained more phosphocreatine than slowly contracting ones. Leaving Nazi Germany in 1933, Nachmansohn moved to Paris where he discovered that acetylcholinesterase is present at extremely high concentrations in the electric organ of the electric fish (*Torpedo marmorata* and *E. electricus*)**.** In 1942 he moved to Columbia University where he and his group discovered choline acetyltransferase, the first biosynthetic enzyme of a neurotransmitter (here acetylcholine), purified acetylcholine esterase and developed the isolated electroplaque [6]. He became member of the Academia Leopoldina in 1963 and of the National Academy of Sciences (USA) in 1965.

## 3. The Techniques or Models Applied and the Reasons for the Failures

Carlos Chagas Filho and his collaborators, who in 1957 had used both a radioactive curare-like substance: “flaxedil” (initially synthesized at the Pasteur Institute by Bovet in 1951) and aqueous extracts of electric tissue of the electric eel *Electrophorus* [4]. Also, Ehrenpreis in the Nachmansohn laboratory demonstrated in 1959 that radioactive curare bound to a soluble protein from the electric tissue of the electric eel [7]. At relatively high ionic strengths, close to the physiological ones, decamethonium, a typical nAChR agonist, did not displace curare from its complex with the protein. In both cases the lack of specificity of the ligands and the conditions of the binding essays were responsible for the failures. De Robertis and colleagues were aware that the receptor could be an integral membrane protein and therefore used a variety of radioactive cholinergic ligands to characterize a “proteolipid” extracted from the electric tissue by a chloroform-methanol mixture. These early claims were subsequently withdrawn by Chagas in 1959 and Ehrenpreis in 1964, and seriously challenged by Levinson and Keynes in 1972, Potter in 1973 and Briley and Changeux in 1977. The final decisive test was to use covalently labeled nAChR (see below) which was not recovered as a proteolipid in chloroform-methanol solutions (see Barrantes et al. [8]).

## 4. The Strategies which Lead to the Identification of the nAChR

The diverse strategies which converged to the first isolation and the *bona fide* identification of the physiological nAChR are summarized as follows:

### 4.1. Allosteric Protein

**Figure 1:**
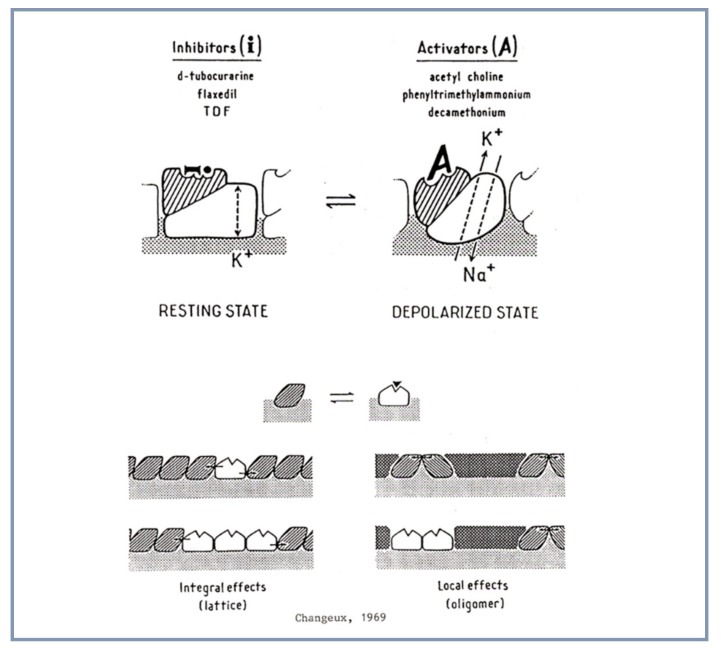
Diagrammatic representation of the then plausible extension of the model of allosteric interaction to the nAChR (reproduced from [18]).

Since Langley’s proposal, the hypothetical receptor molecule was compared by physiologists [9] and others to an enzyme such as acetylcholinesterase yet without proposing a mechanism for the opening of the ion channel. Nachmansohn suggested in 1955 a “change in configuration (conformation) of the protein” [6]. Meanwhile, biochemical investigations on bacterial regulatory enzymes [10,11,12,13] and on hemoglobin [14] offered novel theoretical and experimental models to investigate the elementary molecular mechanisms of biological regulation. They assumed that the interaction between acetylcholine and the ion channel is indirect or *allosteric* and mediated by “a discrete reversible alteration of the molecular structure of the protein or allosteric transition” [15,16,17] (Figure 1).

### 4.2. Electric Organ: the Electroplaque and the Excitable Microsacs

**Figure 2:**
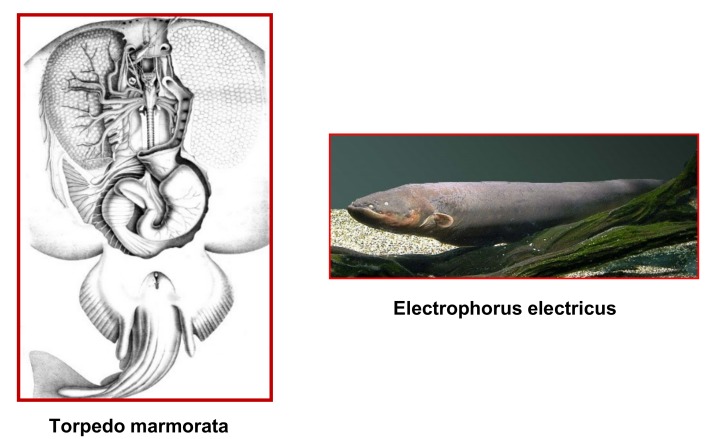
The electric fishes *T. marmorata* and *E. electricus*. In the left part of the figure the electric organs from *T. marmorata* have been uncovered by dissection.

Nachmansohn recognized as early as 1936 the extraordinarily rich content of nicotinic synapses of the fish electric organ [6] (Figure 2). With Ernest Schoffeniels, he devised a method for preparing individual electroplaques, from the same electric organ [19] thus offering the opportunity to investigate, simultaneously, the electrophysiological, pharmacological, and biochemical characteristics of the response to ACh with the same biological system.

**Figure 3:**
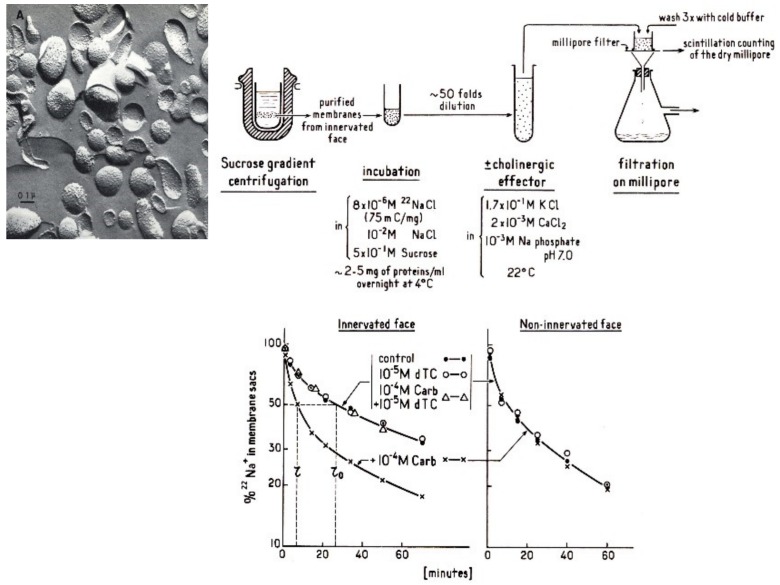
Schematic representation of the microsacs ion flux method (reproduced from Ref [22]).

Crude preparations of electric organ homogenate were available, though very heterogeneous. To simplify the system, Changeux introduced a new approach based upon the electron microscopy of membrane fragments purified from *E. electricus* electric tissue (on the basis of their high content of acetylcholinesterase) which revealed that the majority of the fragments reseal into closed vesicles or “microsacs” [20]. As shown by Kasai and Changeux [21] such microsacs retain permeant ions like radioactive Na^+^ or K^+^ and using a simple filtration technique nAChR agonists caused an increase of the rate of ion flux while the “apparent volume” of the microsacs population did not change (Figure 3).

The response reproduced the postsynaptic response to ACh in a totally acellular system, in the absence of an energy supply and in a chemically defined environment, an observation fully consistent with a spontaneous allosteric mechanism for the opening of the ion channel.

### 4.3. Search for Specific Ligands: Affinity Labeling

**Figure 4:**
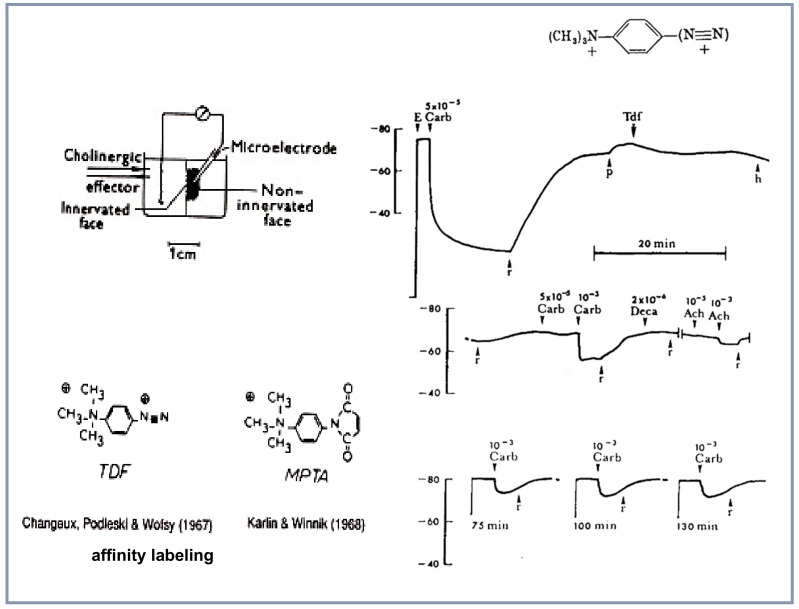
The method of affinity labeling of the nAChR using *p*-(trimethylammonium) benzene diazonium difluoroborate (TDF) with *E. electricus* electroplaque (reproduced from [27]).

Aware of the non-specific binding of curare and flaxedil (Chagas and Ehrenpreis) and in particular to acetylcholinesterase and its allosteric sites [23], the method of affinity labeling designed in the sixties by Jon Singer at the University of California San Diego to label noncatalytic sites, such as those of immunoglobulins was introduced [24]. Affinity labels display both a high affinity for the site to which they bind and a chemically reactive group. The affinity labeling reagents display an enhanced rate of covalent bonding of the reactive group to the target site [24]. The first neurotransmitter receptor explored with an affinity labeling reagent was the muscarinic receptor from smooth muscle by Gill and Rang in 1966 [25], but further identification of the receptor protein appeared difficult and this line of research was abandoned. The first affinity labeling reagent utilized in vivo with the eel electroplaque was *p-*(trimethylamonium) benzene diazonium difluoroborate (TDF) (a compound designed by Fenton and Singer in 1965 to affinity-label immunoglobulin sites [26]] because of its direct relationships with the nicotinic agonist phenyltrimethylammonium (Figure 4). Together with Podleski, Changeux tested it with the electroplaque while in David Nachmansohn’s laboratory at Columbia University: it behaved as expected, as an irreversible competitive antagonist and its effect was protected by D-tubocurarine [27].

Later in 1979 the same molecule TDF was successfully used by Weiland and colleagues [28] to selectively label the nicotinic receptor site *in vitro* with the purified protein and subsequently its dimethyl photo-activable homolog led to the identification of critical amino acids of the ACh binding site [29].

Soon after the experiences with TDF on eel electroplaque [27], an improvement in the affinity labeling for the ACh receptor site was developped by Karlin in 1968 in the Nachmansohn laboratory with a compound designed by Karlin and Winnick [30], where the diazonium group on the phenylmethyl ammonium moiety of TDF was replaced by a maleimide. The compound was named 4-(*N*-maleimido)phenyltrimethylammonium iodide (MPTA). Its selectivity was enhanced because it reacted covalently with the ACh site exclusively when the electroplaque had been exposed to the disulfide reducing agent dithiothreitol [31]. Several new affinity ligands were designed on this basis together with Silman. Attempts to use radiolabeled MPTA to count nAChR sites in the electroplaque appeared encouraging [32], but as stated by Karlin himself, and as in the case of TDF the “specificity was not complete” (p. 260) to efficiently pursue the identification of the receptor. Yet, years later, MPTA successfully led to the identification of the first amino acid from the acetylcholine binding site [33].

### 4.4. Microsacs and Equilibrium Dialysis

**Figure 5:**
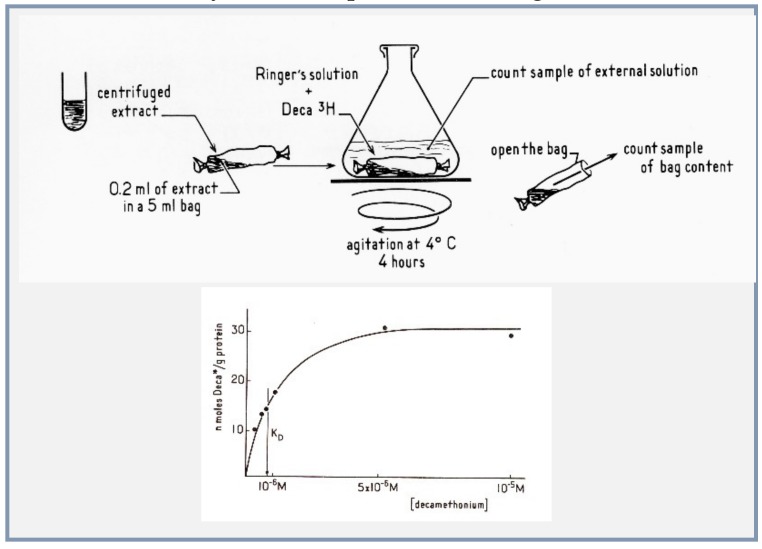
Application of the equilibrium dialysis method of Gilbert and Müller-Hill to isolate the nAChR top method (as described in [34]), bottom binding data (reproduced from [35]).

The decisive step for the *bona fide* isolation and identification of the receptor in a physiological state arose from the joint use of an accurate method of equilibrium binding and–most of all—of an exquisite label of the receptor site. Having purified excitable microsacs from *E. electricus* which respond in vitro to ACh like the native postsynaptic membrane [21], Changeux proposed that the best method to identify the receptor protein in its native, binding conformation was to follow directly the binding of cholinergic ligands by the method of equilibrium dialysis, which was successfully used by Gilbert and Müller-Hill to identify the lac-repressor [34] (Figure 5).

The following experimental conditions for the equilibrium dialysis experiments were selected:

(1) The nicotinic agonist decamethonium was used as the radioactive ligand;
(2) Conditions of solubilization of the electric organ membranes were found with the detergent deoxycholate, to gently extract the membrane proteins without denaturing them;
(3) Concentrations of proteins in the dialysis bag were made high enough to expect a measurable displacement of the ligand by a population of sites in the range of that of the acetylcholinesterase active sites;
(4) Bound decamethonium was displaced by various nicotinic agonists and antagonists, including curare and Flaxedil in the order of their physiological effects [35,36].

### 4.5. Snake Venom Toxins

**Figure 6:**
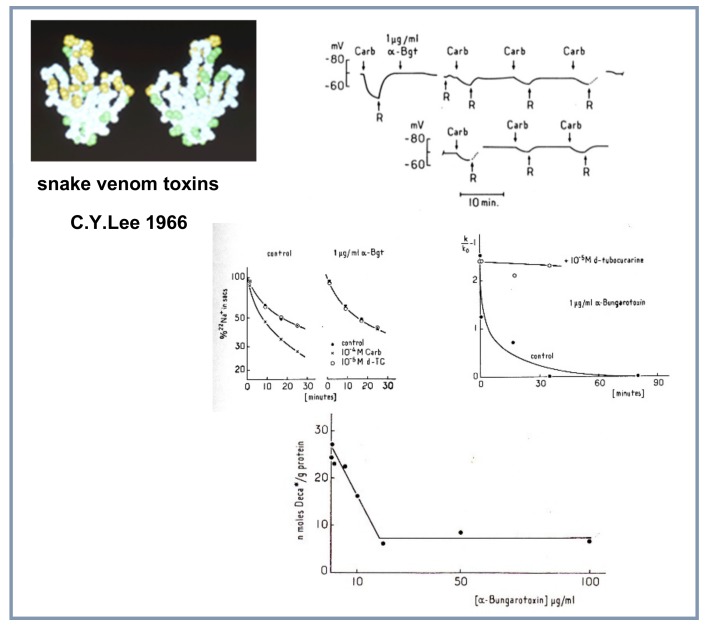
The use of the snake venom toxin α-bungarotoxin to identify the nAChR from the electroplaque (top), the excitable microsacs (middle) and the detergent solubilized extract (bottom) (Reproduced from [36]).

Last, Chen-Yuan Lee, a Taiwanese pharmacologist, a pioneer in the identification of toxins from snake venoms, had found that a toxin from the venom of *Bungarus multicinctus*, α-bungarotoxin (α-BGT), specifically blocks in vivo neuromuscular transmission in high vertebrates at the postsynaptic level without interacting with AChE [37]. Aware of Claude Bernard’s suggestion to use toxic compounds as chemical lancets, Changeux asked Lee, who unexpectedly visited his laboratory at the Institut Pasteur, for a sample of the toxin. A few days later, he received it and immediately tried it in the three systems just mentioned. The result reported by Changeux, Kasai and Lee in 1970 was remarkable [36] and was immediately communicated by Jacques Monod to the Proceedings of the National Academy of Sciences USA (July 30, 1970). αBGT blocked the *E.electricus* electroplaque electrical response in vivo and the microsac ion flux response to nicotinic agonists in vitro; αBGT also blocked the binding of radioactive decamethonium to the detergent extract [35,36] (Figure 6).

This extract contained a protein, sensitive to pronase digestion, that bound nicotinic agonists and the snake venom toxin in a mutually exclusive manner. This nAChR molecule was shown to be a high molecular weight hydrophobic protein that could be physically separated from AChE [35,36,38] as initially predicted by Nachmansohn and by Karlin.

Independently, at University College London, Miledi, Molinoff and Potter [39] attempted to isolate the receptor from a different electric fish *T. marmorata* and Lee’s α-BGT. Their paper came out in *Nature* (received January 28, 1971, published February 19, 1971) months after the initial publication of Changeux and colleagues [35] (published in *Compte Rendus Acad Sc*. 8 June 1970). Unexpectedly, Miledi’s physiological data were not as convincing as those obtained with *E. electrophorus*, possibly because of the difficulty of recording from *T. marmorata* stacks of electroplaques. Isolated electroplaques were not easily available from the *Torpedo* electric organ at that time, but the authors used radioactive I^l31^ labeled bungarotoxin which according to them, would selectively bind to the receptor in its resting state. The most positive aspect of their contributions was the filtration on Sephadex and ultracentrifugation of the toxin-receptor complex following solubilization by 1.5% Triton x 100. The separation between acetylcholinesterase and the toxin binding component was also evident from their work. It was an additional useful step in the early characterization of the receptor protein.

### 4.6. Purification

**Figure 7:**
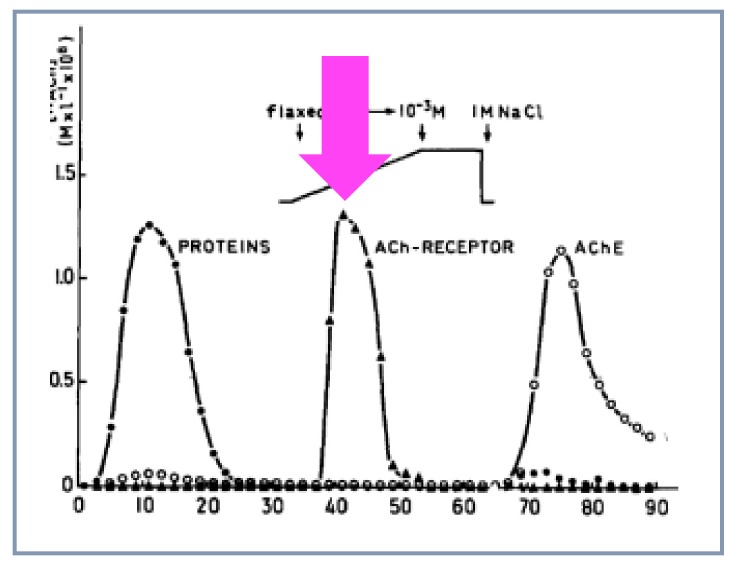
Purification of the nAChR from *E.electricus* the peak of purified nAChR is indicated by a purple arrow (reproduced from [43]).

Further characterization and purification of the receptor protein was helped by the development of another kind of technology. The α-toxin from the black-necked spitting cobra *Naja nigricollis* was covalently coupled to sepharose beads following the method by Porath without losing its immunogenic activity. Mixing these α-toxin beads with the membrane extract revealed that 75-100% of the receptor protein bound to the α-toxin beads while 85-100% of AChE remained in the supernatant. The data published by Meunier and colleagues (*Compte-rendus* January 4, 1971) [38] confirmed the original intuition of Chen-Yuan Lee but, also, introduced in the AChR field a novel technique: affinity chromatography, initially designed by Cuatrecasas. Many groups throughout the world became aware of these new possibilities and actively entered the field. The method was indeed found efficient to purify the receptor protein by Heilbronn [40], Reich [41] and Lindstrom and Patrick [42] (Patrick was a postdoctoral fellow in Changeux’s laboratory when Chen-Yuan Lee visited the Pasteur Institute). Alternative affinity columns were also used with immobilized quaternary ammonium agonists or antagonists by ourselves [43,44] (Figure 7), Raftery [45] and others. All the groups used the snake venom α-toxin to assay the receptor protein.

## 5. First Visualisation of the Receptor Protein Structure by Electron Microscopy

**Figure 8:**
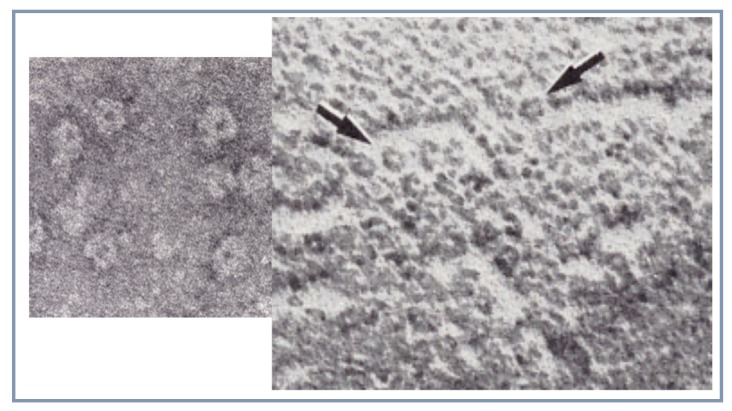
First EM visualisation of the AChR protein: *left* detergent purified AChR protein from *E.electricus* and *right* AChR-rich membranes from *T. marmorata*. The arrows indicate possible pentameric structures (reproduced from [47]).

The nAChR protein purified from *E. electricus* by Olsen et al. [43] and the purified nAChR-rich membranes from *T. marmorata* [46] were examined by electron microscopy by Cartaud in the Changeux team and revealed ring-like particles (8–9 nm in diameter) with a hydrophilic core (about 1.5 nm) linked to a compact bundle [47]. Made up of several (approximately five) subunits, they formed closely packed two-dimensional assemblies in *T. marmorata* postsynaptic membranes [47,48]. These nAChR images of the purified *E electricus* receptor molecule and of the postsynaptic membranes of Torpedo were the first ever published of the structure of a neurotransmitter receptor (Figure 8). They were subsequently described in greater details by Brisson (a former PhD student of Changeux) who discovered that the receptor makes 2-dimensional crystals with aging suspensions of nAChR-rich membranes from *T. marmorata* and subsequently moved in the laboratory of Unwin [49] as postdoctoral fellow.

## 6. Experimental Myasthenia Gravis with Purified nAChR

Patrick and Lindstrom in 1973 [50] injected rabbits with nAChR purified from the electric organ of *E. electricus* emulsified in complete Freund’s adjuvant. It soon resulted in the production of precipitating antibody to nAChR. Then, after the second injection of antigen, the animals developed a flaccid paralysis and abnormal electromyographs characteristic of neuromuscular blockade. Treatment with the anticholinesterases edrophonium or neostigmine dramatically alleviated the paralysis and the fatigue seen in electromyography. This experimental autoimmune myasthenia gravis has become the best animal model of the human disease myasthenia gravis [51]. Patrick and Lindstrom experiment further confirmed the critical role plaid by the isolated receptor in neuromuscular transmission in vivo.

## 7. Primary and Quaternary Structure

The amount of purified nAChR was sufficient to identify the subunit organization of the protein. A first study in Changeux laboratory using partial cross-linking of the purified *E. electricus* nAChR revealed five well defined bands, suggesting a pentameric organization [52]. The pentameric organization was rapidly established by the teams of Karlin and Raftery, who, in addition, discovered that the nAChR molecule is composed of four distinct categories of subunits α, β, γ and δ with slight differences in molecular mass that assemble into an hetero-pentameric oligomer [33,53,54,55,56]. Nothing was known about the chemistry of the subunits. 

However, with the recently developed new technology of high resolution microsequencing, amino acid sequences could be determined from small quantities of protein. The sequence of 20 amino acids comprising the N-terminal domain of the α-subunit of the *T. marmorata* receptor was first established in Changeux’s laboratory [57]. A chemical identity card of the receptor was made available to the scientific community, the first ever established for a neurotransmitter receptor. It was subsequently confirmed in the Raftery laboratory with the α-subunit of *Torpedo californica* [58] and extended to the N-terminal sequence of the four subunits, revealing a number of sequence identities among the subunits [59]. The receptor protein is an authentic oligomer, but pseudo-symmetrical, with a 5-fold axis of rotation perpendicular to the plane of the postsynaptic membrane.

Knowledge of the initial sequence data paved the way to recombinant DNA technologies. The teams of Numa [60,61,62], Heinemann [63,64], Barnard [65] and Changeux [66,67] struggled to clone the complementary DNAs and establish their complete sequence of the different subunits of the nAChR from electric organ and muscle. 

Experiments by Barnard and Miledi had demonstrated that messenger RNA extracted from the electric organ of *Torpedo* injected into *Xenopus* oocytes led to the synthesis and incorporation of functional AChRs into the membrane of the oocyte [68]. Injection of the four mRNAs transcribed from the cloned cDNAs yielded functional nAChRs [69], confirming earlier biochemical reconstitution experiments [70,71] demonstrating that an assembly of the four types of subunits suffices to recover a fully operational nAChR.

Examination of the complete cDNA sequences revealed several common structural domains along the sequences of the subunits that led to the first model of transmembrane organization of nAChR subunits [62,63,64,67]. It was proposed that the long hydrophilic N-terminal segment, four hydrophobic stretches, and a short hydrophilic segment were organized into an extracellular (synaptic) domain, four (transmembrane) α-helices, and an intracellular (cytoplasmic) domain. 

In 1986 and after, closely homologous sequences and the organization of the subunits, including a Cys loop, were found in neuronal nicotinic ACh receptors, including α7- and α4β2-nAChRs [72,73,74], GABA_A_, glycine, 5-HT3 receptors and glutamate-gated chloride channel (GluCl), thus creating the superfamily of pentameric receptors. The recent discovery of cationic orthologs in prokaryotes [75,76] has extended the superfamily, plunging its evolutionary origins back 3 billion years [77].

## 8. The Acetylcholine Nicotinic Receptors to-Day: Biological Functions, Relationships with Brain Diseases and Pharmacology 

The isolation of the nicotinic receptor and the demonstration of its allosteric properties were followed by the identification of many members of the pentameric receptor family in the brain (see [72,73,74]) as well as of the hundreds of receptors from other families, including the vast GPCRs, and olfactory receptors family, the tyrosine kinase receptors and also the popular channel rhodopsin light-gated cation channel. These studies also pioneered the elucidation at the atomic level of the signal transduction mechanism mediated by these receptors and their molecular dynamics [18]. In a general manner, the available experimental data favor, but with possible exceptions, the allosteric scheme of pre-existing conformational equilibria [22,78,79]. The discovery of the acetylcholine receptor as a model of brain receptor brings a new insight about the chemistry of brain communications up to their relationships with higher brain functions [79,80].

From a drug design perspective, the experimental data and their modelling suggest that the design of drugs should be targeted to site(s) present on defined conformation(s) or the receptor molecule rather than to a single category of rigid binding sites, resulting in the production of agonistic *versus* antagonistic ligand. Specifically, in the case of nAChRs, nicotine itself and veranicline, a new drug on the market, are used against tabagism and many nicotinic drugs are currently under clinical tests against Alzheimer depression, schizophrenia, Parkinson, ADHD and pain [81].

The data obtained in vivo with genetically modified mice on nicotine addiction lead to the identification of nAChRs subunit combinations mediating the rewarding effects of nicotine administration. These studies on the molecular basis of nicotine addiction [82] offer a novel panel of conformational targets for long-term smoking cessation therapies and possibly for alcohol consumption.

The concept of allosteric modulators as pharmacological agents binding to allosteric sites distinct from the principal biological site for neurotransmitters and/or hormones, strikingly differs from the classical concept of competitive drug binding. Without a doubt, the notion of allosteric modulation has created a major landmark in the strategies of drug design for ligand-gated ion channels but also GPCRs, resulting in the successful development of new classes of drugs used in the clinic. Allosteric modulators include some of the most commonly prescribed psychopharmaceutical drug in the world, such as benzodiazepines, barbiturates, local and general anesthetics, and several new families of drugs [83] also important allosteric drugs used in clinic as Gleevec (allosteric inhibitor of Abl tyrosine kinase), Cinacalcet (allosteric activator of calcium-sensing receptor) and Maraviroc (allosteric inhibitor of chemokine receptor). This understanding has major practical consequences in the conception of new pharmacological agents at both orthosteric and allosteric modulatory sites in each of their conformational states [18]. Attesting to the growing interest in allosteric phenomena, the Allosteric database 2019 lists 82 070 entries as allosteric modulators, among them 538 allosteric drugs, with at least 91 currently used as medicines. A new pharmacology is born.

The concept of receptor diseases as caused, for instance, by gain (or loss) of function mutations of neurotransmitter receptors as a result of the stabilization of distinct allosteric states. These diseases include, in the case of nAChRs, congenital myasthenia gravis [83]), autosomal dominant frontal lobe nocturnal epilepsy [84] but also multiple genetic diseases affecting different categories of receptors such as GPCRs and tyrosine kinase receptors (including oncogenes). The Allosteric database 2019 lists 5983 allostery-related diseases.

These investigations further document and enrich what may be called a ‘chemical theory of higher brain functions’. Within this framework, all processes at the multiple levels of organization that span the human brain, from synapse to consciousness, rest upon a biochemical universe of allosteric transitions that mediate neuronal and interneuronal communications [79,80].

## 9. International Recognition and Importance for the History of Neuroscience

The structural identification of the nAChR as the first membrane receptor for a neurotransmitter involved several laboratories throughout the world. Yet, its first biochemical identification from fish electric organ, was made in Europe. Subsequently several receptors were isolated and identified for different neurotransmitters again in Europe: the glycine receptor by Betz in 1982, the GABA_A_ receptor by Barnard in 1982 and Seeburg in 1986, then in the United States, brain nAChRs were identified by Patrick and Heinemann in 1986, the glutamate receptors by Heinemann in 1989, the G-Protein-Coupled. Receptors by Levitsky and by Lefkowitz in 1974, including the olfactory receptors by Buck and Axel in 1991 and many others.

David Nachmansohn wrote to Changeux (letter dated November 29 1970 (*Archives Institut Pasteur*) “I am very excited by your great success with the receptor using alpha-bungarotoxin. I just read your publication in the PNAS. It is truly magnificent! Your success opens a new chapter of molecular neurobiology”.
